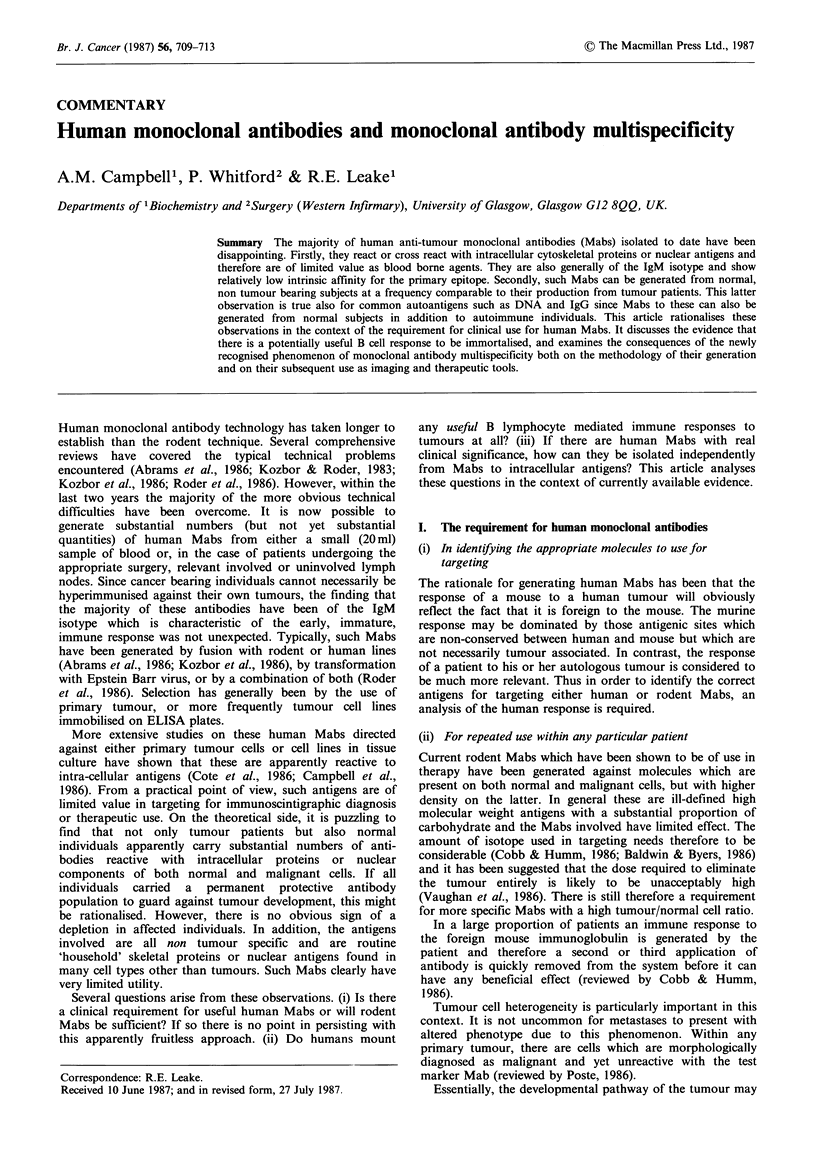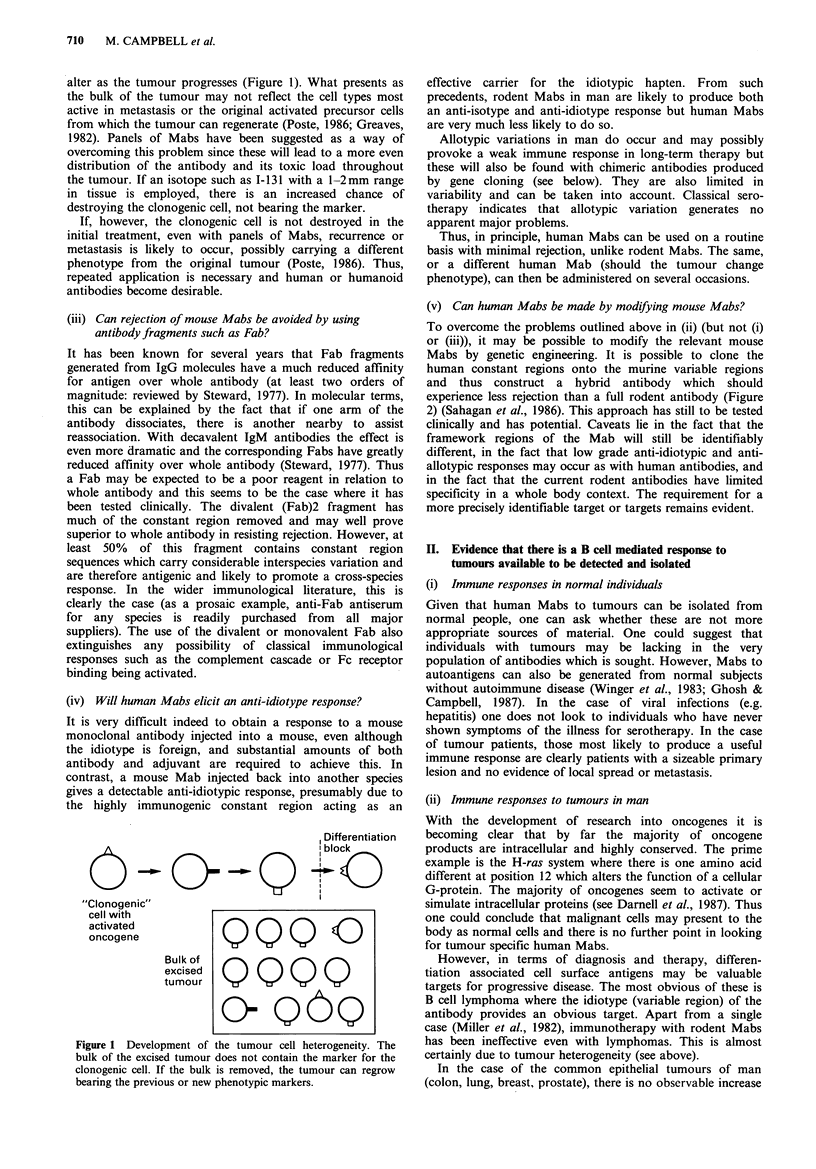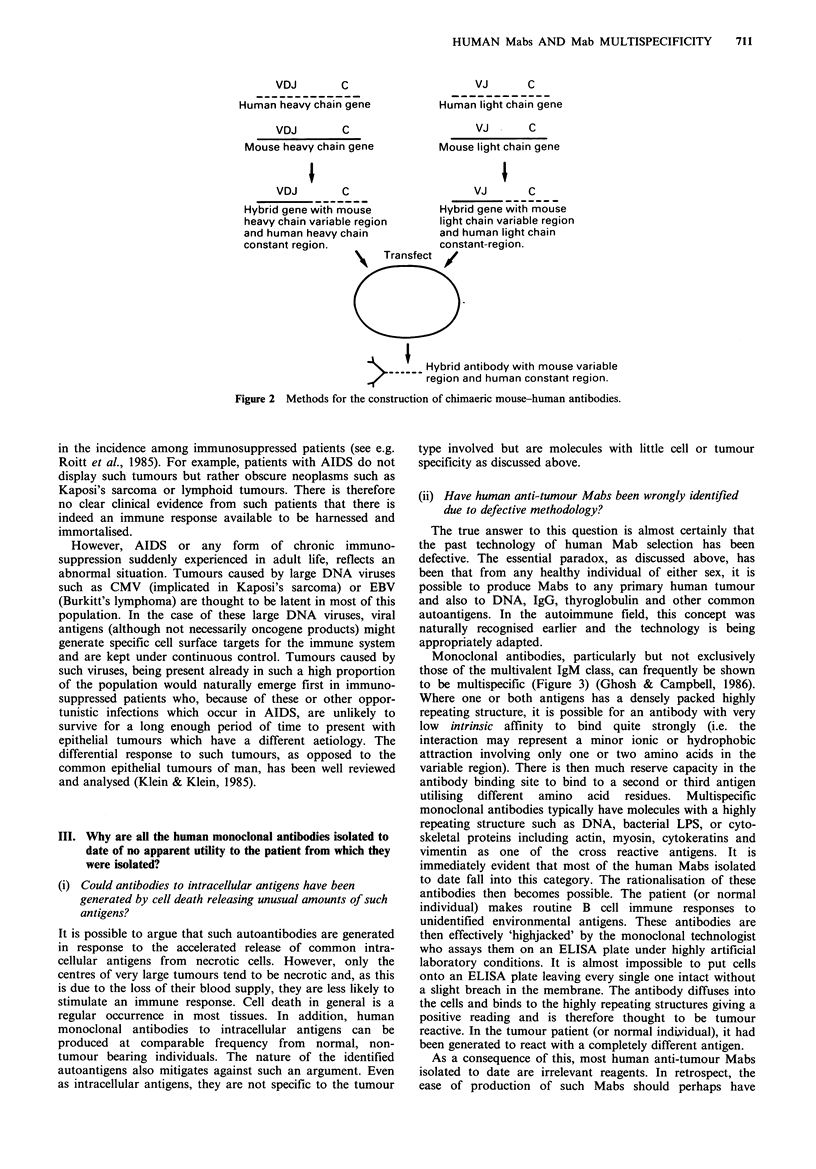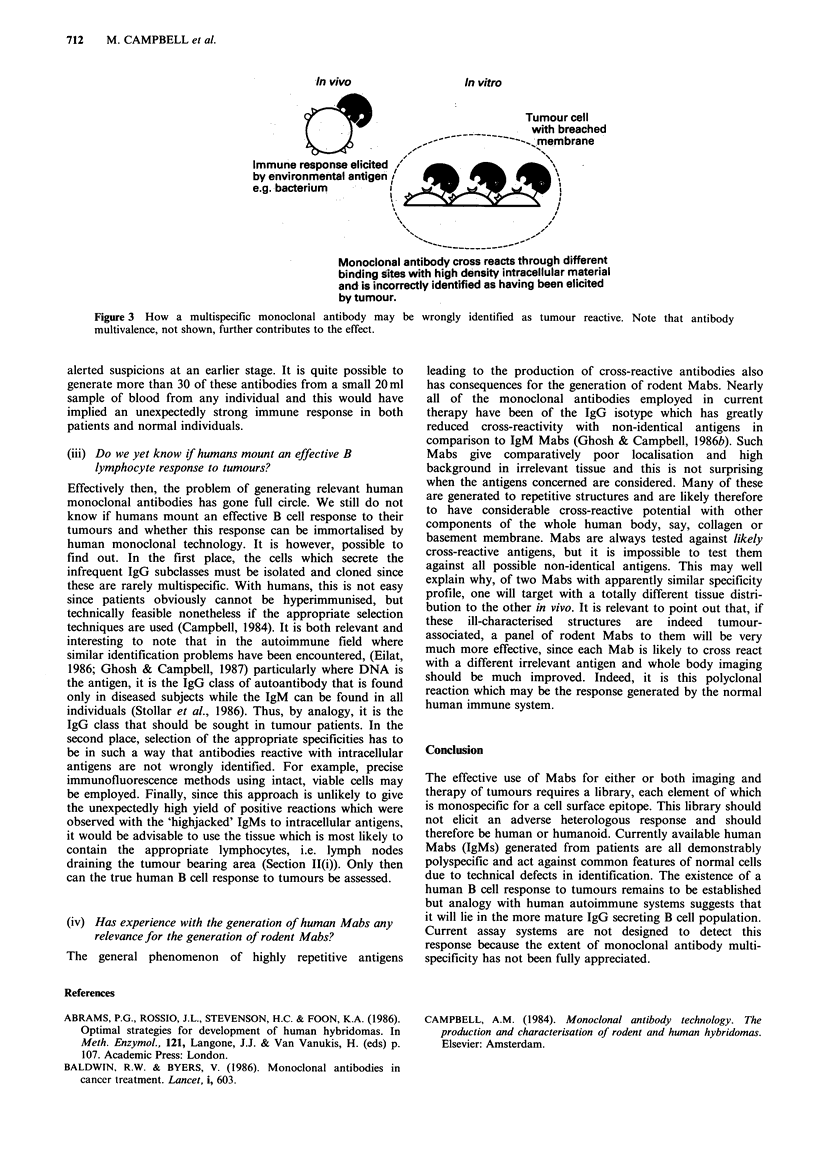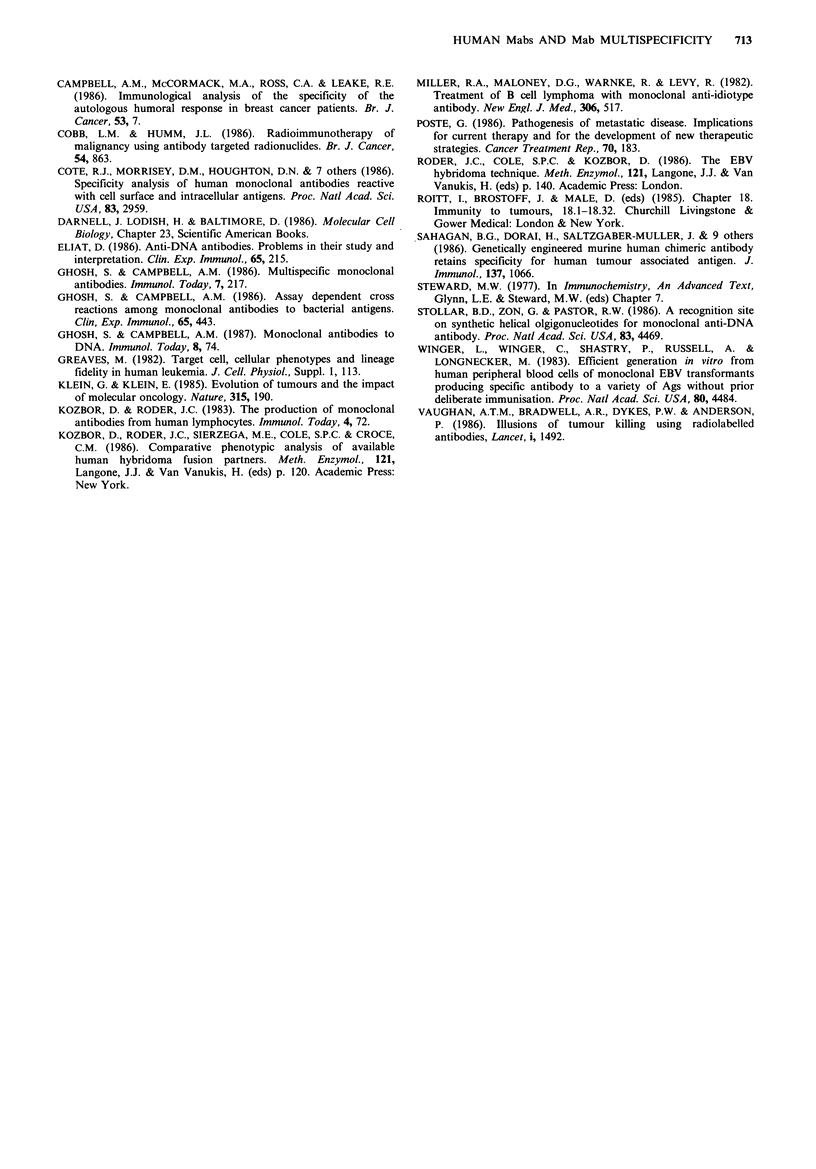# Human monoclonal antibodies and monoclonal antibody multispecificity.

**DOI:** 10.1038/bjc.1987.275

**Published:** 1987-12

**Authors:** A. M. Campbell, P. Whitford, R. E. Leake

**Affiliations:** Department of Biochemistry, University of Glasgow, UK.

## Abstract

The majority of human anti-tumour monoclonal antibodies (Mabs) isolated to date have been disappointing. Firstly, they react or cross react with intracellular cytoskeletal proteins or nuclear antigens and therefore are of limited value as blood borne agents. They are also generally of the IgM isotype and show relatively low intrinsic affinity for the primary epitope. Secondly, such Mabs can be generated from normal, non tumour bearing subjects at a frequency comparable to their production from tumour patients. This latter observation is true also for common autoantigens such as DNA and IgG since Mabs to these can also be generated from normal subjects in addition to autoimmune individuals. This article rationalises these observations in the context of the requirement for clinical use for human Mabs. It discusses the evidence that there is a potentially useful B cell response to be immortalised, and examines the consequences of the newly recognised phenomenon of monoclonal antibody multispecificity both on the methodology of their generation and on their subsequent use as imaging and therapeutic tools.


Br. J. Cancer (1987) 56, 709–713 © The Macmillan Press Ltd., 1987

COMMENTARY

### Human monoclonal antibodies and monoclonal antibody multispecificity

A.M. Campbell[1], P. Whitford[2] & R.E. Leake[1]

*Departments of [1]Biochemistry and [2]Surgery (Western Infirmary), University of Glasgow, Glasgow G12 8QQ, UK.*

**Summary** The majority of human anti-tumour monoclonal antibodies (Mabs) isolated to date have been disappointing. Firstly, they react or cross react with intracellular cytoskeletal proteins or nuclear antigens and therefore are of limited value as blood borne agents. They are also generally of the IgM isotype and show relatively low intrinsic affinity for the primary epitope. Secondly, such Mabs can be generated from normal, non tumour bearing subjects at a frequency comparable to their production from tumour patients. This latter observation is true also for common autoantigens such as DNA and IgG since Mabs to these can also be generated from normal subjects in addition to autoimmune individuals. This article rationalises these observations in the context of the requirement for clinical use for human Mabs. It discusses the evidence that there is a potentially useful B cell response to be immortalised, and examines the consequences of the newly recognised phenomenon of monoclonal antibody multispecificity both on the methodology of their generation and on their subsequent use as imaging and therapeutic tools.

Human monoclonal antibody technology has taken longer to establish than the rodent technique. Several comprehensive reviews have covered the typical technical problems encountered (Abrams *et al.*, 1986; Kozbor & Roder, 1983; Kozbor *et al.*, 1986; Roder *et al.*, 1986). However, within the last two years the majority of the more obvious technical difficulties have been overcome. It is now possible to generate substantial numbers (but not yet substantial quantities) of human Mabs from either a small (20 ml) sample of blood or, in the case of patients undergoing the appropriate surgery, relevant involved or uninvolved lymph nodes. Since cancer bearing individuals cannot necessarily be hyperimmunised against their own tumours, the finding that the majority of these antibodies have been of the IgM isotype which is characteristic of the early, immature, immune response was not unexpected. Typically, such Mabs have been generated by fusion with rodent or human lines (Abrams *et al.*, 1986; Kozbor *et al.*, 1986), by transformation with Epstein Barr virus, or by a combination of both (Roder *et al.*, 1986). Selection has generally been by the use of primary tumour, or more frequently tumour cell lines immobilised on ELISA plates.

More extensive studies on these human Mabs directed against either primary tumour cells or cell lines in tissue culture have shown that these are apparently reactive to intra-cellular antigens (Cote *et al.*, 1986; Campbell *et al.*, 1986). From a practical point of view, such antigens are of limited value in targeting for immunoscintigraphic diagnosis or therapeutic use. On the theoretical side, it is puzzling to find that not only tumour patients but also normal individuals apparently carry substantial numbers of antibodies reactive with intracellular proteins or nuclear components of both normal and malignant cells. If all individuals carried a permanent protective antibody population to guard against tumour development, this might be rationalised. However, there is no obvious sign of a depletion in affected individuals. In addition, the antigens involved are all *non* tumour specific and are routine 'household' skeletal proteins or nuclear antigens found in many cell types other than tumours. Such Mabs clearly have very limited utility.

Several questions arise from these observations. (i) Is there a clinical requirement for useful human Mabs or will rodent Mabs be sufficient? If so there is no point in persisting with this apparently fruitless approach. (ii) Do humans mount any *useful* B lymphocyte mediated immune responses to tumours at all? (iii) If there are human Mabs with real clinical significance, how can they be isolated independently from Mabs to intracellular antigens? This article analyses these questions in the context of currently available evidence.

### I. The requirement for human monoclonal antibodies

### (i) *In identifying the appropriate molecules to use for targeting*

The rationale for generating human Mabs has been that the response of a mouse to a human tumour will obviously reflect the fact that it is foreign to the mouse. The murine response may be dominated by those antigenic sites which are non-conserved between human and mouse but which are not necessarily tumour associated. In contrast, the response of a patient to his or her autologous tumour is considered to be much more relevant. Thus in order to identify the correct antigens for targeting either human or rodent Mabs, an analysis of the human response is required.

### (ii) *For repeated use within any particular patient*

Current rodent Mabs which have been shown to be of use in therapy have been generated against molecules which are present on both normal and malignant cells, but with higher density on the latter. In general these are ill-defined high molecular weight antigens with a substantial proportion of carbohydrate and the Mabs involved have limited effect. The amount of isotope used in targeting needs therefore to be considerable (Cobb & Humm, 1986; Baldwin & Byers, 1986) and it has been suggested that the dose required to eliminate the tumour entirely is likely to be unacceptably high (Vaughan *et al.*, 1986). There is still therefore a requirement for more specific Mabs with a high tumour/normal cell ratio.

In a large proportion of patients an immune response to the foreign mouse immunoglobulin is generated by the patient and therefore a second or third application of antibody is quickly removed from the system before it can have any beneficial effect (reviewed by Cobb & Humm, 1986).

Tumour cell heterogeneity is particularly important in this context. It is not uncommon for metastases to present with altered phenotype due to this phenomenon. Within any primary tumour, there are cells which are morphologically diagnosed as malignant and yet unreactive with the test marker Mab (reviewed by Poste, 1986).

Essentially, the developmental pathway of the tumour may

Correspondence: R.E. Leake.
Received 10 June 1987; and in revised form, 27 July 1987.

alter as the tumour progresses (Figure 1). What presents as the bulk of the tumour may not reflect the cell types most active in metastasis or the original activated precursor cells from which the tumour can regenerate (Poste, 1986; Greaves, 1982). Panels of Mabs have been suggested as a way of overcoming this problem since these will lead to a more even distribution of the antibody and its toxic load throughout the tumour. If an isotope such as I-131 with a 1–2 mm range in tissue is employed, there is an increased chance of destroying the clonogenic cell, not bearing the marker.

If, however, the clonogenic cell is not destroyed in the initial treatment, even with panels of Mabs, recurrence or metastasis is likely to occur, possibly carrying a different phenotype from the original tumour (Poste, 1986). Thus, repeated application is necessary and human or humanoid antibodies become desirable.

### (iii) *Can rejection of mouse Mabs be avoided by using antibody fragments such as Fab?*

It has been known for several years that Fab fragments generated from IgG molecules have a much reduced affinity for antigen over whole antibody (at least two orders of magnitude: reviewed by Steward, 1977). In molecular terms, this can be explained by the fact that if one arm of the antibody dissociates, there is another nearby to assist reassociation. With decavalent IgM antibodies the effect is even more dramatic and the corresponding Fabs have greatly reduced affinity over whole antibody (Steward, 1977). Thus a Fab may be expected to be a poor reagent in relation to whole antibody and this seems to be the case where it has been tested clinically. The divalent (Fab)2 fragment has much of the constant region removed and may well prove superior to whole antibody in resisting rejection. However, at least 50% of this fragment contains constant region sequences which carry considerable interspecies variation and are therefore antigenic and likely to promote a cross-species response. In the wider immunological literature, this is clearly the case (as a prosaic example, anti-Fab antiserum for any species is readily purchased from all major suppliers). The use of the divalent or monovalent Fab also extinguishes any possibility of classical immunological responses such as the complement cascade or Fc receptor binding being activated.

### (iv) *Will human Mabs elicit an anti-idiotype response?*

It is very difficult indeed to obtain a response to a mouse monoclonal antibody injected into a mouse, even although the idiotype is foreign, and substantial amounts of both antibody and adjuvant are required to achieve this. In contrast, a mouse Mab injected back into another species gives a detectable anti-idiotypic response, presumably due to the highly immunogenic constant region acting as an

**Figure 1** Development of the tumour cell heterogeneity. The bulk of the excised tumour does not contain the marker for the clonogenic cell. If the bulk is removed, the tumour can regrow bearing the previous or new phenotypic markers.

effective carrier for the idiotypic hapten. From such precedents, rodent Mabs in man are likely to produce both an anti-isotype and anti-idiotype response but human Mabs are very much less likely to do so.

Allotypic variations in man do occur and may possibly provoke a weak immune response in long-term therapy but these will also be found with chimeric antibodies produced by gene cloning (see below). They are also limited in variability and can be taken into account. Classical serotherapy indicates that allotypic variation generates no apparent major problems.

Thus, in principle, human Mabs can be used on a routine basis with minimal rejection, unlike rodent Mabs. The same, or a different human Mab (should the tumour change phenotype), can then be administered on several occasions.

### (v) *Can human Mabs be made by modifying mouse Mabs?*

To overcome the problems outlined above in (ii) (but not (i) or (iii)), it may be possible to modify the relevant mouse Mabs by genetic engineering. It is possible to clone the human constant regions onto the murine variable regions and thus construct a hybrid antibody which should experience less rejection than a full rodent antibody (Figure 2) (Sahagan et al., 1986). This approach has still to be tested clinically and has potential. Caveats lie in the fact that the framework regions of the Mab will still be identifiably different, in the fact that low grade anti-idiotypic and anti-allotypic responses may occur as with human antibodies, and in the fact that the current rodent antibodies have limited specificity in a whole body context. The requirement for a more precisely identifiable target or targets remains evident.

### II. Evidence that there is a B cell mediated response to tumours available to be detected and isolated

### (i) *Immune responses in normal individuals*

Given that human Mabs to tumours can be isolated from normal people, one can ask whether these are not more appropriate sources of material. One could suggest that individuals with tumours may be lacking in the very population of antibodies which is sought. However, Mabs to autoantigens can also be generated from normal subjects without autoimmune disease (Winger et al., 1983; Ghosh & Campbell, 1987). In the case of viral infections (e.g. hepatitis) one does not look to individuals who have never shown symptoms of the illness for serotherapy. In the case of tumour patients, those most likely to produce a useful immune response are clearly patients with a sizeable primary lesion and no evidence of local spread or metastasis.

### (ii) *Immune responses to tumours in man*

With the development of research into oncogenes it is becoming clear that by far the majority of oncogene products are intracellular and highly conserved. The prime example is the H-*ras* system where there is one amino acid different at position 12 which alters the function of a cellular G-protein. The majority of oncogenes seem to activate or simulate intracellular proteins (see Darnell et al., 1987). Thus one could conclude that malignant cells may present to the body as normal cells and there is no further point in looking for tumour specific human Mabs.

However, in terms of diagnosis and therapy, differentiation associated cell surface antigens may be valuable targets for progressive disease. The most obvious of these is B cell lymphoma where the idiotype (variable region) of the antibody provides an obvious target. Apart from a single case (Miller et al., 1982), immunotherapy with rodent Mabs has been ineffective even with lymphomas. This is almost certainly due to tumour heterogeneity (see above).

In the case of the common epithelial tumours of man (colon, lung, breast, prostate), there is no observable increase

**Figure 2** Methods for the construction of chimaeric mouse–human antibodies.

in the incidence among immunosuppressed patients (see e.g. Roitt *et al.*, 1985). For example, patients with AIDS do not display such tumours but rather obscure neoplasms such as Kaposi's sarcoma or lymphoid tumours. There is therefore no clear clinical evidence from such patients that there is indeed an immune response available to be harnessed and immortalised.

However, AIDS or any form of chronic immunosuppression suddenly experienced in adult life, reflects an abnormal situation. Tumours caused by large DNA viruses such as CMV (implicated in Kaposi's sarcoma) or EBV (Burkitt's lymphoma) are thought to be latent in most of this population. In the case of these large DNA viruses, viral antigens (although not necessarily oncogene products) might generate specific cell surface targets for the immune system and are kept under continuous control. Tumours caused by such viruses, being present already in such a high proportion of the population would naturally emerge first in immunosuppressed patients who, because of these or other opportunistic infections which occur in AIDS, are unlikely to survive for a long enough period of time to present with epithelial tumours which have a different aetiology. The differential response to such tumours, as opposed to the common epithelial tumours of man, has been well reviewed and analysed (Klein & Klein, 1985).

### III. Why are all the human monoclonal antibodies isolated to date of no apparent utility to the patient from which they were isolated?

### (i) *Could antibodies to intracellular antigens have been generated by cell death releasing unusual amounts of such antigens?*

It is possible to argue that such autoantibodies are generated in response to the accelerated release of common intracellular antigens from necrotic cells. However, only the centres of very large tumours tend to be necrotic and, as this is due to the loss of their blood supply, they are less likely to stimulate an immune response. Cell death in general is a regular occurrence in most tissues. In addition, human monoclonal antibodies to intracellular antigens can be produced at comparable frequency from normal, non-tumour bearing individuals. The nature of the identified autoantigens also mitigates against such an argument. Even as intracellular antigens, they are not specific to the tumour type involved but are molecules with little cell or tumour specificity as discussed above.

### (ii) *Have human anti-tumour Mabs been wrongly identified due to defective methodology?*

The true answer to this question is almost certainly that the past technology of human Mab selection has been defective. The essential paradox, as discussed above, has been that from any healthy individual of either sex, it is possible to produce Mabs to any primary human tumour and also to DNA, IgG, thyroglobulin and other common autoantigens. In the autoimmune field, this concept was naturally recognised earlier and the technology is being appropriately adapted.

Monoclonal antibodies, particularly but not exclusively those of the multivalent IgM class, can frequently be shown to be multispecific (Figure 3) (Ghosh & Campbell, 1986). Where one or both antigens has a densely packed highly repeating structure, it is possible for an antibody with very low *intrinsic* affinity to bind quite strongly (i.e. the interaction may represent a minor ionic or hydrophobic attraction involving only one or two amino acids in the variable region). There is then much reserve capacity in the antibody binding site to bind to a second or third antigen utilising different amino acid residues. Multispecific monoclonal antibodies typically have molecules with a highly repeating structure such as DNA, bacterial LPS, or cytoskeletal proteins including actin, myosin, cytokeratins and vimentin as one of the cross reactive antigens. It is immediately evident that most of the human Mabs isolated to date fall into this category. The rationalisation of these antibodies then becomes possible. The patient (or normal individual) makes routine B cell immune responses to unidentified environmental antigens. These antibodies are then effectively 'highjacked' by the monoclonal technologist who assays them on an ELISA plate under highly artificial laboratory conditions. It is almost impossible to put cells onto an ELISA plate leaving every single one intact without a slight breach in the membrane. The antibody diffuses into the cells and binds to the highly repeating structures giving a positive reading and is therefore thought to be tumour reactive. In the tumour patient (or normal individual), it had been generated to react with a completely different antigen.

As a consequence of this, most human anti-tumour Mabs isolated to date are irrelevant reagents. In retrospect, the ease of production of such Mabs should perhaps have

**Figure 3** How a multispecific monoclonal antibody may be wrongly identified as tumour reactive. Note that antibody multivalence, not shown, further contributes to the effect.

alerted suspicions at an earlier stage. It is quite possible to generate more than 30 of these antibodies from a small 20 ml sample of blood from any individual and this would have implied an unexpectedly strong immune response in both patients and normal individuals.

(iii) *Do we yet know if humans mount an effective B lymphocyte response to tumours?*

Effectively then, the problem of generating relevant human monoclonal antibodies has gone full circle. We still do not know if humans mount an effective B cell response to their tumours and whether this response can be immortalised by human monoclonal technology. It is however, possible to find out. In the first place, the cells which secrete the infrequent IgG subclasses must be isolated and cloned since these are rarely multispecific. With humans, this is not easy since patients obviously cannot be hyperimmunised, but technically feasible nonetheless if the appropriate selection techniques are used (Campbell, 1984). It is both relevant and interesting to note that in the autoimmune field where similar identification problems have been encountered, (Eilat, 1986; Ghosh & Campbell, 1987) particularly where DNA is the antigen, it is the IgG class of autoantibody that is found only in diseased subjects while the IgM can be found in all individuals (Stollar *et al.*, 1986). Thus, by analogy, it is the IgG class that should be sought in tumour patients. In the second place, selection of the appropriate specificities has to be in such a way that antibodies reactive with intracellular antigens are not wrongly identified. For example, precise immunofluorescence methods using intact, viable cells may be employed. Finally, since this approach is unlikely to give the unexpectedly high yield of positive reactions which were observed with the 'highjacked' IgMs to intracellular antigens, it would be advisable to use the tissue which is most likely to contain the appropriate lymphocytes, i.e. lymph nodes draining the tumour bearing area (Section II(i)). Only then can the true human B cell response to tumours be assessed.

(iv) *Has experience with the generation of human Mabs any relevance for the generation of rodent Mabs?*

The general phenomenon of highly repetitive antigens leading to the production of cross-reactive antibodies also has consequences for the generation of rodent Mabs. Nearly all of the monoclonal antibodies employed in current therapy have been of the IgG isotype which has greatly reduced cross-reactivity with non-identical antigens in comparison to IgM Mabs (Ghosh & Campbell, 1986*b*). Such Mabs give comparatively poor localisation and high background in irrelevant tissue and this is not surprising when the antigens concerned are considered. Many of these are generated to repetitive structures and are likely therefore to have considerable cross-reactive potential with other components of the whole human body, say, collagen or basement membrane. Mabs are always tested against *likely* cross-reactive antigens, but it is impossible to test them against all possible non-identical antigens. This may well explain why, of two Mabs with apparently similar specificity profile, one will target with a totally different tissue distribution to the other *in vivo*. It is relevant to point out that, if these ill-characterised structures are indeed tumour-associated, a panel of rodent Mabs to them will be very much more effective, since each Mab is likely to cross react with a different irrelevant antigen and whole body imaging should be much improved. Indeed, it is this polyclonal reaction which may be the response generated by the normal human immune system.

### Conclusion

The effective use of Mabs for either or both imaging and therapy of tumours requires a library, each element of which is monospecific for a cell surface epitope. This library should not elicit an adverse heterologous response and should therefore be human or humanoid. Currently available human Mabs (IgMs) generated from patients are all demonstrably polyspecific and act against common features of normal cells due to technical defects in identification. The existence of a human B cell response to tumours remains to be established but analogy with human autoimmune systems suggests that it will lie in the more mature IgG secreting B cell population. Current assay systems are not designed to detect this response because the extent of monoclonal antibody multispecificity has not been fully appreciated.

### References

ABRAMS, P.G., ROSSIO, J.L., STEVENSON, H.C. & FOON, K.A. (1986). Optimal strategies for development of human hybridomas. In *Meth. Enzymol.*, **121**, Langone, J.J. & Van Vanukis, H. (eds) p. 107. Academic Press: London.

BALDWIN, R.W. & BYERS, V. (1986). Monoclonal antibodies in cancer treatment. *Lancet*, **i**, 603.

CAMPBELL, A.M. (1984). *Monoclonal antibody technology. The production and characterisation of rodent and human hybridomas.* Elsevier: Amsterdam.

CAMPBELL, A.M., McCORMACK, M.A., ROSS, C.A. & LEAKE, R.E. (1986). Immunological analysis of the specificity of the autologous humoral response in breast cancer patients. *Br. J. Cancer*, **53**, 7.

COBB, L.M. & HUMM, J.L. (1986). Radioimmunotherapy of malignancy using antibody targeted radionuclides. *Br. J. Cancer*, **54**, 863.

COTE, R.J., MORRISEY, D.M., HOUGHTON, D.N. & 7 others (1986). Specificity analysis of human monoclonal antibodies reactive with cell surface and intracellular antigens. *Proc. Natl Acad. Sci. USA*, **83**, 2959.

DARNELL, J. LODISH, H. & BALTIMORE, D. (1986). *Molecular Cell Biology*, Chapter 23, Scientific American Books.

ELIAT, D. (1986). Anti-DNA antibodies. Problems in their study and interpretation. *Clin. Exp. Immunol.*, **65**, 215.

GHOSH, S. & CAMPBELL, A.M. (1986). Multispecific monoclonal antibodies. *Immunol. Today*, **7**, 217.

GHOSH, S. & CAMPBELL, A.M. (1986). Assay dependent cross reactions among monoclonal antibodies to bacterial antigens. *Clin, Exp. Immunol.*, **65**, 443.

GHOSH, S. & CAMPBELL, A.M. (1987). Monoclonal antibodies to DNA. *Immunol. Today*, **8**, 74.

GREAVES, M. (1982). Target cell, cellular phenotypes and lineage fidelity in human leukemia. *J. Cell. Physiol.*, Suppl. 1, 113.

KLEIN, G. & KLEIN, E. (1985). Evolution of tumours and the impact of molecular oncology. *Nature*, **315**, 190.

KOZBOR, D. & RODER, J.C. (1983). The production of monoclonal antibodies from human lymphocytes. *Immunol. Today*, **4**, 72.

KOZBOR, D., RODER, J.C., SIERZEGA, M.E., COLE, S.P.C. & CROCE, C.M. (1986). Comparative phenotypic analysis of available human hybridoma fusion partners. *Meth. Enzymol.*, **121**, Langone, J.J. & Van Vanukis, H. (eds) p. 120. Academic Press: New York.

MILLER, R.A., MALONEY, D.G., WARNKE, R. & LEVY, R. (1982). Treatment of B cell lymphoma with monoclonal anti-idiotype antibody. *New Engl. J. Med.*, **306**, 517.

POSTE, G. (1986). Pathogenesis of metastatic disease. Implications for current therapy and for the development of new therapeutic strategies. *Cancer Treatment Rep.*, **70**, 183.

RODER, J.C., COLE, S.P.C. & KOZBOR, D. (1986). The EBV hybridoma technique. *Meth. Enzymol.*, **121**, Langone, J.J. & Van Vanukis, H. (eds) p. 140. Academic Press: London.

ROITT, I., BROSTOFF, J. & MALE, D. (eds) (1985). Chapter 18. Immunity to tumours, 18.1–18.32. Churchill Livingstone & Gower Medical: London & New York.

SAHAGAN, B.G., DORAI, H., SALTZGABER-MULLER, J. & 9 others (1986). Genetically engineered murine human chimeric antibody retains specificity for human tumour associated antigen. *J. Immunol.*, **137**, 1066.

STEWARD, M.W. (1977). In *Immunochemistry, An Advanced Text*, Glynn, L.E. & Steward, M.W. (eds) Chapter 7.

STOLLAR, B.D., ZON, G. & PASTOR, R.W. (1986). A recognition site on synthetic helical olgigonucleotides for monoclonal anti-DNA antibody. *Proc. Natl Acad. Sci. USA*, **83**, 4469.

WINGER, L., WINGER, C., SHASTRY, P., RUSSELL, A. & LONGNECKER, M. (1983). Efficient generation *in vitro* from human peripheral blood cells of monoclonal EBV transformants producing specific antibody to a variety of Ags without prior deliberate immunisation. *Proc. Natl Acad. Sci. USA*, **80**, 4484.

VAUGHAN, A.T.M., BRADWELL, A.R., DYKES, P.W. & ANDERSON, P. (1986). Illusions of tumour killing using radiolabelled antibodies, *Lancet*, **i**, 1492.